# Efficient Delivery of Gemcitabine by Estrogen Receptor-Targeted PEGylated Liposome and Its Anti-Lung Cancer Activity In Vivo and In Vitro

**DOI:** 10.3390/pharmaceutics15030988

**Published:** 2023-03-19

**Authors:** Huan Tang, Zheng Zhang, Ming Zhu, Yizhuo Xie, Zhe Lv, Rui Liu, Yujia Shen, Jin Pei

**Affiliations:** Department of Biopharmacy, School of Pharmaceutical Sciences, Jilin University, Changchun 130021, China; tanghuan20@mails.jlu.edu.cn (H.T.); 18686615926@163.com (Z.Z.); mingzhu21@mails.jlu.edu.cn (M.Z.); xieyz21@mails.jlu.edu.cn (Y.X.); lvzhe19@mails.jlu.edu.cn (Z.L.); liurui20@mails.jlu.edu.cn (R.L.); shenyj20@mails.jlu.edu.cn (Y.S.)

**Keywords:** gemcitabine, PEGylated liposome, estrogen receptor, targeting efficiency, lung cancer

## Abstract

Lung cancer is one of the main causes of cancer-related deaths. At present, the main treatment method for lung cancer is chemotherapy. Gemcitabine (GEM) is widely applied in lung cancer treatment, but its lack of targeting ability and serious side effects limit its application. In recent years, nanocarriers have become the focus of research to solve the above problems. Here, we prepared estrone (ES)-modified GEM-loaded PEGylated liposomes (ES-SSL-GEM) for enhanced delivery by identifying the overexpressed estrogen receptor (ER) on lung cancer A549 cells. We studied the characterization, stability, release behavior, cytotoxicity, targeting ability, endocytosis mechanism, and antitumor ability to prove the therapeutic effect of ES-SSL-GEM. The results showed that ES-SSL-GEM presented a uniform particle size of 131.20 ± 0.62 nm, a good stability, and a slowly released behavior. Moreover, ES-SSL-GEM enhanced tumor-targeting ability, and the endocytosis mechanism studies confirmed that the ER-mediated endocytosis had the most crucial effect. Furthermore, ES-SSL-GEM had the best inhibitory effect on A549 cell proliferation and significantly suppressed the tumor growth in vivo. These results suggest that ES-SSL-GEM is a promising agent for treating lung cancer.

## 1. Introduction

In the past hundred years, lung cancer has changed from a rarely occurring disease to a leading cause of cancer-related death in humans [1]. Globally, lung cancer cases and deaths are increasing, accounting for 2.09 million new cases and 1.76 million deaths in 2018, which represented 11.6% of total cancer cases and 18.4% of total cancer deaths [2]. Non-small cell lung cancer (NSCLC), which accounts for nearly 80% of lung cancers, is the most common category of lung cancer [3]. Therefore, the treatment of NSCLC has become the top priority target in human lung cancer.

The stage of tumor development is an important factor in determining the treatment of lung cancer [4]. At present, besides surgical resection of cancerous lesions at the early stage, the main treatment methods are chemotherapy and radiotherapy, of which chemotherapy is the preferred treatment for advanced lung cancer [5]. Commonly used first-line chemotherapy drugs for lung cancer mainly include cisplatin, gemcitabine (GEM), vinorelbine, or taxanes (paclitaxel or docetaxel) [6]. These chemotherapy drugs have comparable efficacy in patients with NSCLC and can prolong the survival period of patients. In addition, all the chemo-drugs have different toxicity profiles [7,8]. Among them, GEM, a pyrimidine analogue of the nucleoside antimetabolite, also known as 2′,2′-difluorodeoxycytidine, has been commonly used in the treatment of NSCLC for many years [9,10]. GEM, as a pro-drug, needs to undergo three steps of phosphorylation into tumor cells to transform into active components, which are responsible for producing cytotoxic functions [11,12]. However, GEM also has some limitations in its application, mainly because of its short half-life, poor metabolic stability, and fast elimination rate in the body [13,14]. Therefore, a high dose of continuous intravenous administration is necessary to maintain an effective drug concentration, which also brings toxicities, including neutropenia and myelosuppression [15,16]. In order to provide a full therapeutic effect of GEM, it has become more important to develop a suitable nanocarrier to improve the delivery of GEM to the targeted tumor and maintain a high drug concentration for a longer time, so as to reduce the medicinal dose of the drug.

At present, a variety of nanocarriers have attracted wide attention due to their passive or active tumor-targeted efficiency, such as liposomes [17], micelles [18], dendrimers [19], and nanoparticles [20]. Among them, liposomes, composed of phospholipids and cholesterol, have demonstrated a variety of advantages, including ameliorating drug pharmacokinetics and improving the safety to the human normal tissues [21,22]. However, conventional liposomes are easily recognized by the reticuloendothelial system (RES), resulting in the liposomes being quickly removed [23]. To solve this problem, polyethylene glycol (PEG) is usually modified on liposomes to increase the steric stability and reduce phagocytosis by the RES [24,25]. Therefore, PEGylated liposomes can prolong the half-life [26]. Another disadvantage of conventional liposomes is the lack of specific targeting [27]. The obstacle could be overcome in combination with modifying ligands that specifically and actively target the tumor cells, such as polymers, peptides, or antibodies [28,29]. Estrogen receptors (ERs) were identified in the late 1960s and have two subtypes, ERα and ERβ, which exist in breasts, uteruses, ovaries and other organs [30]. Recent studies have shown that the expression of ERs in NSCLCs is much higher than that in normal lung tissue [31,32]. Therefore, the use of ER ligand estrone (ES) to target ERs on the surface of NSCLCs can achieve the purpose of targeted treatment of NSCLCs [33].

In this study, an ER-targeted PEGylated liposome for the delivery of GEM (ES-SSL-GEM) was designed. This nanocarrier exhibited the properties of sustained release by PEG coating and facilitating cellular uptake via the recognition between ES-conjugated liposome and ER overexpressed on the A549 cell membrane. The characterization, target efficiency, and anti-tumor ability were evaluated and compared with conventional liposomes. ES-SSL-GEM had excellent tumor-targeting and anti-tumor abilities. In conclusion, utilizing this liposomal formulation to deliver GEM presented promising therapeutic effects on NSCLC treatment.

## 2. Materials and Methods

### 2.1. Materials

ES-PEG_2000_-DSPE was synthesized by our laboratory as described previously [34]. Gemcitabine hydrochloride was purchased from Meilun Biotechnology (Dalian, China). Rhodamine B (Rh B) and soybean lecithin was obtained from Tianjin Guangfu fine chemical Research Institute (Tianjin, China). The 1,2-distearoyl-sn-glycero-3-phosphoethanolamine-N- [methoxy (poly-ethylene glycol) 2000] (mPEG_2000_-DSPE) and fetal bovine serum (FBS) were purchased from Shanghai Xibao Biotechnology Co., Ltd. (Shanghai, China). Cholesterol was purchased from Shanghai Huishi biochemical reagent Co., Ltd. (Shanghai, China). DMEM and MEM mediums were purchased from Gibco (Beijing, China). 3-(4,5-dimethylthiazol-2-yl)-2,5-diphenyltetrazolium bromide (MTT) was purchased from Beijing Mengyimei Biotechnology Co., Ltd. (Beijing, China).

### 2.2. Cell Lines and Animals

Human NSCLC cell line A549 and murine fibroblast cell line L929 were bought from Procell Life Science (Wuhan, China). A 10% FBS and 1% penicillin–streptomycin solution was added to DMEM to form a culture medium for A549 cells. A 10% FBS and 1% penicillin-streptomycin solution was added to MEM to form a culture medium for L929 cells. Cell culture was performed at 37 °C and 5% CO_2_. The 5–6-week-old SPF BALB/c nude mice were bought from the Beijing Vital River Laboratory Animal Technology Co., Ltd. (Beijing, China). The experimental animals were free to get food and water, and they were fed in the laboratory center at 25 °C, 60% humidity, and a 12 h dark/light cycle.

### 2.3. Preparation of GEM Liposomes

The ES-SSL-GEM was prepared using the ammonium sulfate gradient method. Soybean lecithin, cholesterol, and mPEG_2000_-DSPE were precisely weighed at a molar ratio of 4:1:0.25 and dissolved in chloroform. The lipid solution was dried into a thin film on a rotary evaporator under a vacuum condition and a 40 °C water bath. It was then hydrated using ammonium sulfate solution, followed by vortex oscillation and ultrasonic for 10 min. Then, the liposomes were extruded through 220 and 80 nm polycarbonate membranes. The liposome was dialyzed overnight using a dialysis bag (MWCO 8.0–14.0 kDa) in 1.0 L normal saline to remove unentrapped ammonium sulfate. The liposomal solution and 0.8 mg GEM were mixed for incubation for 1 h at 37 °C. Finally, ES-SSL-GEM was obtained by incubating the solution of ES-PEG_2000_-DSPE dissolved by ultra-pure water (lipid: ES-PEG_2000_-DSPE = 200:1 molar ratio) with the above liposomes in a 37 °C water bath for 1 h. Free GEM was removed using a dialysis bag (MWCO 8.0–14.0 kDa). Conventional GEM liposomes (L-GEM) were prepared without the use of mPEG_2000_-DSPE and ES-PEG_2000_-DSPE. PEGylated GEM liposomes (SSL-GEM) and ER-targeted GEM liposomes (ES-L-GEM) were also prepared without the use of ES-PEG_2000_-DSPE or mPEG_2000_-DSPE, respectively, according to the same procedure.

### 2.4. Characterization of Liposomes

The dynamic light scattering (DLS) (Malvern Instruments, the UK; Zetasizer Nano ZS90) was used to measure the particle size, zeta potential, and polydispersity index (PDI) of different liposome formulations.

The morphology of SSL-GEM and ES-SSL-GEM was photographed using transmission electron microscopy (TEM) (JEM-2100F, Tokyo, Japan). After three folds dilutions, the liposome sample was carefully dropped on the carbon-coated copper grids. After drying, the samples were then stained with 1% phosphotungstic acid solution. After drying the dye with filter paper, ultra-pure water was dropped, and then was dried again using filter paper. After natural drying, the liposomes were photographed.

The encapsulation efficiency (EE) of GEM separated by dialysis was determined using a UV spectrophotometer [35]. The liposome was added into the dialysis bag (MWCO 8.0–14.0 kDa), which was immersed in PBS and stirred slowly for 4 h to completely remove the free GEM. After dialysis, the 100.0 μL liposome samples were taken out and added to 1.9 mL of 1.0% SDS for sonication for 10 min. The absorbance was determined at 268 nm wavelength using a UV spectrophotometer. The following Equation (1) was applied to calculate the EE:

EE (%) = (W_G_/W_0_) × 100% (1)

where W_G_ was the amount of GEM entrapped in liposomes and W_0_ was the total amount of GEM added.

### 2.5. In Vitro Stability of Liposomes

In order to evaluate the stability of liposomes, the four kinds of liposome formulations were stored at 4 °C and 25 °C. Partial liposomes were taken out at given time intervals. The free GEM was removed using the method of characterization of liposomes. The concentration of GEM was determined using a UV spectrophotometer, and was recorded as C_n_ (n = 1, 2, 4, 8, 24 and 48 h). C_0_ was the concentration of GEM after dialysis at 0 h. The following Equation (2) was applied to calculate the leakage rate of GEM:

Leakage rate = [1 − (C_n_/C_0_)] × 100% (2)

In addition, the long-term stability of ES-SSL-GEM was further investigated. ES-SSL-GEM was stored at 4 °C, 25 °C, and 37 °C for a week. The particle size, zeta potential, and leakage rate of ES-SSL-GEM were measured at preset time intervals.

### 2.6. Drug Release Behavior

Drug release experiments of GEM liposome formulations were performed via the dialysis method. A total of 1.0 mL of the liposome sample or GEM solution was added into a dialysis bag (MWCO 8.0–14.0 kDa) and immersed in 20.0 mL of pH 7.4 PBS at 37 °C, accompanied by slight stirring. At predetermined time points, 1.0 mL release medium was taken out, while 1.0 mL fresh release medium was added. The concentrations of GEM were determined using a UV spectrophotometer at 268 nm.

### 2.7. Cytotoxicity Assay

In vitro cytotoxicity of the liposomes was evaluated using the MTT assay in A549 cells. The A549 cells were inoculated with 1.0 × 10^4^ cells/well into a 96-well plate and incubated at 37 °C for 24 h under 5% CO_2_. The cells were exposed to free GEM, L-GEM, ES-L-GEM, SSL-GEM, and ES-SSL-GEM at predetermined GEM concentrations. After 24 h, 48 h, or 72 h of co-incubation, 20.0 μL of 5.0 mg/mL MTT solution was put into each well and remained with the cells for 4 h. Subsequently, 150.0 μL DMSO was used to replace the supernatant, and the mixture continued to shake for another 10 min to dissolve the formazan crystals. A microplate reader (DNM-9602, Pu Lang, Beijing, China) was used to measure the optical density (OD) at 490 nm. The IC_50_ was calculated using GraphPad Prism 5.0 (GraphPad Software, San Diego, CA, USA). The following Equation (3) was applied to calculate the cell viability:

Cell viability (%) = (OD_d_ − OD_b_)/(OD_c_ − OD_b_) × 100% (3)

where OD_d_ represented the OD of the groups treated with different GEM formulations, OD_c_ represented the OD of untreated groups, and OD_b_ represented the OD of the blank formulation.

In order to test the drug delivery system’s toxicity on non-cancerous cells, L929 cells were inoculated in 96-well plates with 5.0 × 10^3^ cells per well. After overnight incubation, cells were treated with Blank-ES-SSL of varying concentrations and incubated for 24, 48, and 72 h. After incubation, cytotoxicity was detected according to the above MTT method.

### 2.8. Cellular Uptake

GEM without fluorescence signals was replaced by Rh B, which exhibited fluorescence signals in this study. ER-targeted PEGylated Rh B-loaded liposome (ES-SSL-Rh B) was prepared using the thin film hydration method. Briefly, soybean lecithin, cholesterol, and mPEG_2000_-DSPE (11:9:1, molar ratio) were added into the rotary evaporation bottle and dissolved using chloroform. Lipid films were formed using the rotary evaporator under vacuum condition at 100 rpm and 40 °C. Then, the PBS containing Rh B was added to hydrate the dry film for 2 h, followed by vortex oscillation. The sample was sonicated for 10 min. The targeted fragment ES-PEG_2000_-DSPE (ES-PEG_2000_-DSPE: lipids = 1:200 molar ratio) was co-incubated with the above liposomes in a 37 °C water bath for 1 h to obtain ES-SSL-Rh B. Conventional Rh B liposomes (L-Rh B) were prepared without the use of mPEG_2000_-DSPE and ES-PEG_2000_-DSPE. PEGylated Rh B liposomes (SSL-Rh B) and ER-targeted Rh B liposomes (ES-L-Rh B) were also prepared without the use of ES-PEG_2000_-DSPE or mPEG_2000_-DSPE, respectively, according to the same procedure.

Uptake of A549 cells for different preparations was evaluated using fluorescence microscopy (Olympus IX71, Tokyo, Japan). Briefly, A549 cells were inoculated in a 24-well plate at a density of 5.0 × 10^4^ cells per well and incubated for 24 h. Then, ES-SSL-Rh B, SSL-Rh B, ES-L-Rh B, and L-Rh B were added at a final Rh B concentration of 5.0 μg/mL. After incubation for 1 h, 2 h, 3 h, and 4 h, the cells were observed under fluorescence microscopy after being washed three times with cold PBS. Image J software was applied to analyze the quantitative results.

### 2.9. Endocytosis Mechanism

To investigate the endocytosis mechanism of ES-SSL-GEM, the competitive inhibitor of ES and several common endocytosis inhibitors of genistein, sucrose, and amiloride were used to block specific endocytic pathways. Briefly, A549 cells were cultured for 24 h in 24-well plates at a density of 5.0 × 10^4^ per well. Cells were pretreated with 0.15 mM ES, 0.45 M sucrose, 0.2 M genistein, 0.01 M amiloride, and PBS as a control for 30 min. Following this, the culture medium was discarded carefully, and the cells were co-incubated with ES-SSL-Rh B for 2 h. Cells were imaged under a fluorescence microscope. Image J software was applied to analyze the quantitative results. In addition, after the A549 cells were blocked with ES, SSL-Rh B was added and incubated with the cells to exclude the effect of ES itself on uptake.

### 2.10. In Vivo Tumor-Targeting Study

DiR, as a fluorescent probe, allows the biodistribution of liposomes in mice to be visualized. ER-targeted PEGylated DiR liposomes (ES-SSL-DiR) were prepared using the film hydration method. The soybean lecithin, cholesterol, mPEG_2000_-DSPE (11:9:1, molar ratio), and DiR were put into a rotary evaporation bottle containing chloroform to form a lipid film, and then PBS was applied to hydrate the lipid film. The molar ratio of lipids to DiR was 1000:1. All subsequent procedures were performed following the steps described in the Rh B liposomes preparations section.

A xenograft mouse model of NSCLC was established to explore the targeted ability of ES-SSL-DiR in vivo. Briefly, 1.0 × 10^7^ cells/0.2 mL of A549 cells were subcutaneously injected into the back of 5–6-week-old BALB/c nude mice. Once the tumor size was approximately 200 mm^3^, the tumor-bearing mice were injected with SSL-DiR and ES-SSL-DiR intravenously. The mice were anesthetized and the biodistribution of fluorescence in mice was monitored using the IVIS spectrum small-animal imaging system (IVIS SPECTRUM, Waltham, MA, USA) after injections at 2 h, 6 h, 12 h, and 24 h. After the imaging was performed, the mice were dissected to harvest major organs and the tumor. The ex vivo fluorescence images were also obtained using the IVIS spectrum small-animal imaging system. Finally, the fluorescence in both living and ex vivo tumors was quantified using the IVIS spectrum small-animal imaging system.

### 2.11. In Vivo Antitumor Efficacy

For the purpose of exploring the in vivo anti-tumor effect, BALB/c nude mice bearing A549 xenograft tumors were established. Briefly, A549 cells suspended in serum-free DMEM medium were inoculated subcutaneously in the right back of the mice. Once the tumor sizes reached 100 mm^3^, mice were injected by tail vein with blank liposome (Blank-ES-SSL), free GEM, SSL-GEM, ES-SSL-GEM, and normal saline, at a dose of 8.0 mg/kg GEM equivalent. All the different formulations were injected twice a week over a period of 24 days. Body weight and tumor volume of mice was recorded every three days to assess the anti-tumor efficacy and the safety of formulations. The tumor volume (V) was measured using vernier calipers and calculated as Equation (4). After 24 days, the tumors were collected and weighed.

V = 1/2 × a × b^2^ (4)

where “a” and “b” represented the maximum and minimum diameters of each tumor, respectively.

The following Equation (5) was applied to calculate the tumor inhibition rate (TIR):

TIR (%) = (1 − W_drug_/W_control_) × 100% (5)

where W_drug_ was the tumor weights of treatment group and W_control_ was the tumor weights of control group, respectively.

### 2.12. Statistical Analysis

All data were presented as mean value ± standard deviation (SD). Statistical comparisons were completed using one-way analysis of variance or Student’s *t*-test, and differences with *p* < 0.05 were considered significant.

## 3. Results and Discussion

### 3.1. Characterization of Liposomes

**Figure 1:**
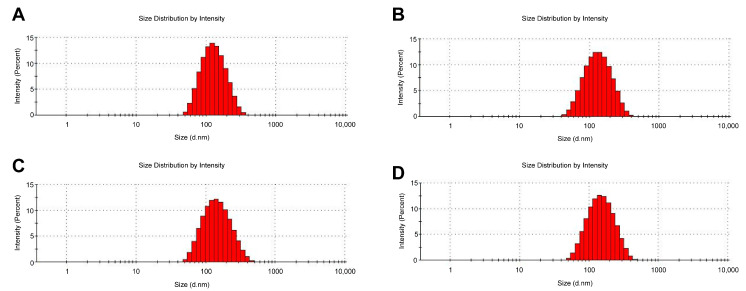
Particle size distribution of GEM liposomes measured by DLS, including (**A**) L-GEM, (**B**) ES-L-GEM, (**C**) SSL-GEM, and (**D**) ES-SSL-GEM.

The characterization results of different GEM liposomes are shown in Table 1, including particle size, PDI, zeta potential, and EE. Particle size is an important factor that affects the physical and chemical characteristics of liposomes. The data showed that the particle size of the four liposomes ranged from 115 nm to 135 nm. The particle sizes of SSL-GEM (129.60 ± 0.68 nm) and ES-SSL-GEM (131.20 ± 0.62 nm) slightly exceeded L-GEM (118.80 ± 0.05 nm) and ES-L-GEM (120.10 ± 0.16 nm) because of the modification of the PEG molecules. However, the modification of the ES-targeting ligand did not significantly increase the particle size of the liposomes, which was probably due to the low concentration of the ES-targeting ligand. More importantly, the particle size of all liposomes did not exceed 200 nm, which indicated that liposomes could be easily gathered at the site of the tumor through the EPR effect [36]. The PDIs of the GEM liposomes were less than 0.20 and, as shown in Figure 1, four liposome formulations also showed narrow and uniform particle size distributions, indicating that the liposome formulations were suitable for intravenous injection. On the other hand, all the liposome formulations exhibited negative zeta potentials, which could improve the stability of liposomes [37]. The zeta potentials of L-GEM, ES-L-GEM, SSL-GEM, and ES-SSL-GEM were −25.67 ± 0.61 mV, −24.97 ± 0.34 mV, −10.04 ± 0.56 mV, and −9.73 ± 0.41 mV, respectively. Among them, the zeta potential of PEGylated liposomes slightly exceeded that of L-GEM and ES-L-GEM, which was caused by the fact that the liposome surface modified with PEG molecules could shield the partial charge of the liposome formulations [23].

For a drug formulation, EE plays a key role in drug delivery. The EEs of GEM for L-GEM and ES-L-GEM were 48.27 ± 3.61% and 48.90 ± 1.73%, respectively. After the introduction of the PEG fragment, the EEs of SSL-GEM and ES-SSL-GEM increased to approximately 55.93 ± 2.98% and 56.35 ± 1.38%, respectively. This could be explained by DSPE-mPEG_2000_ increasing the stability of the lipid layer through the steric effect [38]. However, the addition of the ES-PEG_2000_-DSPE fragment showed almost no influence on the EE of the liposome formulations.

**Figure 2:**
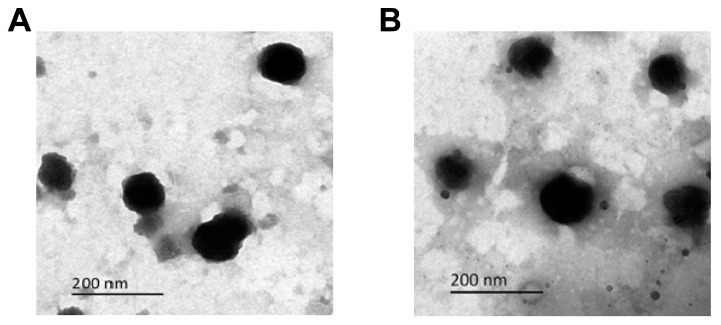
Transmission electron microscopy images of (**A**) SSL-GEM and (**B**) ES-SSL-GEM. Scale bar: 200 nm.

TEM is a commonly used method to visualize the morphology of SSL-GEM and ES-SSL-GEM. As shown in Figure 2A,B, the SSL-GEM and ES-SSL-GEM displayed a spherical shape. The particle size observed using TEM was consistent with the measurement results of DLS. There was no significant difference in particle size between SSL-GEM and ES-SSL-GEM. Previous studies have shown that, compared with other shapes, nanoparticles with the spherical shape were conducive to cellular uptake [39].

### 3.2. In Vitro Stability of Liposomes

**Figure 3:**
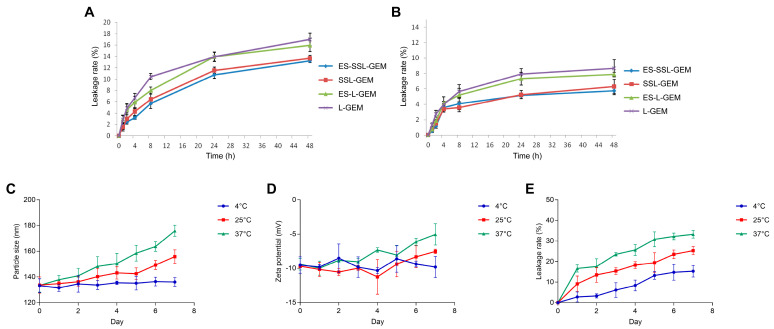
The stability of GEM liposomal formulations. The leakage rate of four GEM liposomes at (**A**) 25 °C and (**B**) 4 °C for 48 h. (**C**) Particle size, (**D**) zeta potential, and (**E**) leakage rate of ES-SSL-GEM stored at 4 °C, 25 °C, and 37 °C for one week.

From the perspective of nanomaterials, the stability of the liposome is an important physi-chemical property of the drug delivery system. Therefore, the leakage rate was detected as an evaluation indicator to access the stability of different GEM liposomes at the storage temperatures of 4 °C and 25 °C for 48 h. As shown in Figure 3A,B, the leakage rates of all four GEM liposomes increased slightly with the extension of time. The leakage rates were less than 18% at 25 °C and 10% at 4 °C within 48 h, indicating that the liposome preparations were relatively stable. It is worth noting that the PEGylated liposomes, including ES-SSL-GEM and SSL-GEM, showed lower leakage rates than ES-L-GEM and L-GEM at 4 °C and 25 °C, proving that the addition of PEG could protect the structural integrity of liposomes through the steric hindrance effect [40]. We continued to investigate the stability of ES-SSL-GEM stored at different temperatures for a week. As shown in Figure 3C–E, the particle size and zeta potential of ES-SSL-GEM did not change significantly at 4 °C, and the leakage rate was less than 20% within a week, indicating that it had good stability. Then, with the increase in storage temperature, the particle size and zeta potential of liposomes changed significantly, and the leakage rate was also higher than that at 4 °C, indicating that temperature would affect the stability of liposomes. This result also suggested that ES-SSL-GEM was more suitable for storage at 4 °C, which was owing to hydrolysis and oxidation of phospholipids influencing the stability of liposomes [41,42]. When the storage temperature was increased, this effect of phospholipids was accelerated, resulting in an increase in the leakage rate.

### 3.3. Drug Release Behavior

**Figure 4:**
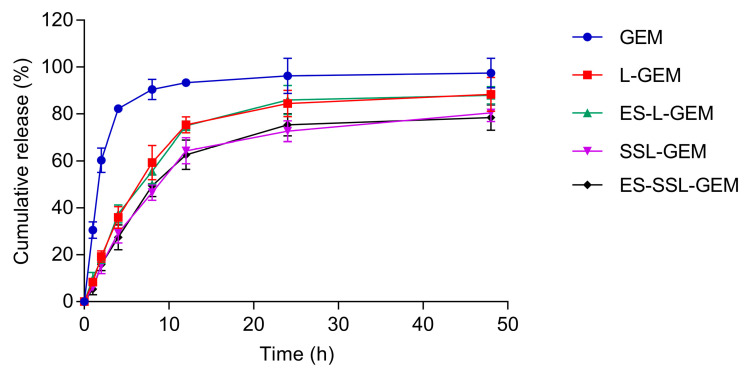
Cumulative release of GEM from the different liposomes in pH 7.4 PBS medium (n = 3).

The release behaviors in vitro of GEM from the liposomes in pH 7.4 PBS medium were measured and are shown in Figure 4. Free GEM showed a rapid release behavior, and the cumulative release rate exceeded 90% at 8 h. The cumulative release rates of L-GEM, ES-L-GEM, SSL-GEM, and ES-SSL-GEM at 48 h were 88.33%, 87.93%, 80.46%, and 78.46%, respectively. All four liposomes showed slow release and sustained behavior, and no burst release phenomenon was observed, which could be due to the lipid bilayer stabilization of the liposome. The release of GEM needed to pass through the lipid bilayer, which could effectively slow down the release of drugs [43]. At the same time, the absence of burst release also indicated that all drugs were stably loaded into the liposomes and no drugs were absorbed onto the carrier surface [44]. In addition, compared with the L-GEM and ES-L-GEM, the release of GEM from SSL-GEM and ES-SSL-GEM was much slower due to the addition of PEG fragments which could increase the stability of liposomes. The excellent release behavior of ES-SSL-GEM could be beneficial to anticancer therapy.

### 3.4. Cytotoxicity Assay

**Figure 5:**
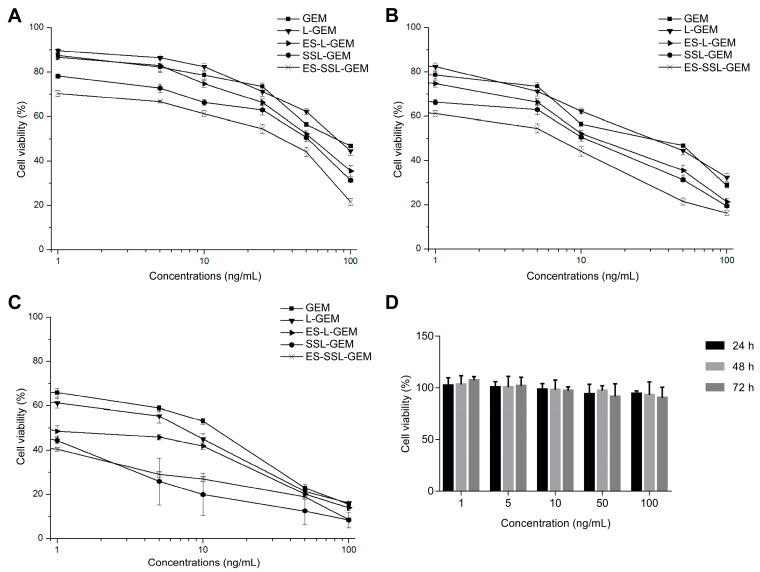
Cell viabilities of A549 cells after incubation with GEM, L-GEM, ES-L-GEM, SSL-GEM, or ES-SSL-GEM for (**A**) 24 h, (**B**) 48 h, and (**C**) 72 h (n = 3). (**D**) Cell viabilities of L929 cells after incubation with Blank-ES-SSL for 24 h, 48 h, and 72 h (n = 3).

The cytotoxicity effects of GEM and GEM liposomal formulations for A549 cells in vitro were evaluated. Results are shown in Figure 5A–C and IC_50_ values are summarized in Table 2. All five GEM formulations exhibited highly dose-dependent cytotoxicity and time-dependent cytotoxicity on A549 cells. The results exhibited that the cytotoxicity of the five GEM formulations for A549 cells from strong to weak were ES-SSL-GEM, SSL-GEM, ES-L-GEM, L-GEM, and GEM. The toxicity of four kinds of GEM liposomes on A549 cells was stronger than that of free GEM because of the high affinity between liposomes and the cell membrane, so GEM encapsulated inside the liposomes could easily enter into the cells to exert an anti-tumor effect. However, ES-SSL-GEM and SSL-GEM showed more cytotoxicity than ES-L-GEM and L-GEM, which proved that the existence of PEG could indeed improve the inhibitory effect of GEM on tumor cells. The inhibitory ability of ES-SSL-GEM was significantly superior to that of other preparations, with the lowest value of 0.09 ± 0.02 ng/mL at 72 h, which was approximately 76.6 times, 11.7 times, and 2.4 times lower than that of L-GEM, ES-L-GEM, and SSL-GEM with the values of 6.89 ± 0.43 ng/mL, 1.05 ± 0.85 ng/mL, and 0.22 ± 0.07 ng/mL, respectively. The experimental results indicated that ES-SSL-GEM had the strongest cytotoxicity against A549 cells, likely due to faster and increased recognition and absorption by A549 cells through the specific ER highly expressed on the A549 cell surface. In addition, it was observed in Figure 5D that Blank-ES-SSL did not cause a significant decrease in cell viability of L929 cells during incubation. Even at the highest concentration, cell viability was still more than 90%, indicating that Blank-ES-SSL was not toxic to normal cells and was a safe drug delivery carrier.

### 3.5. Cellular Uptake

**Figure 6:**
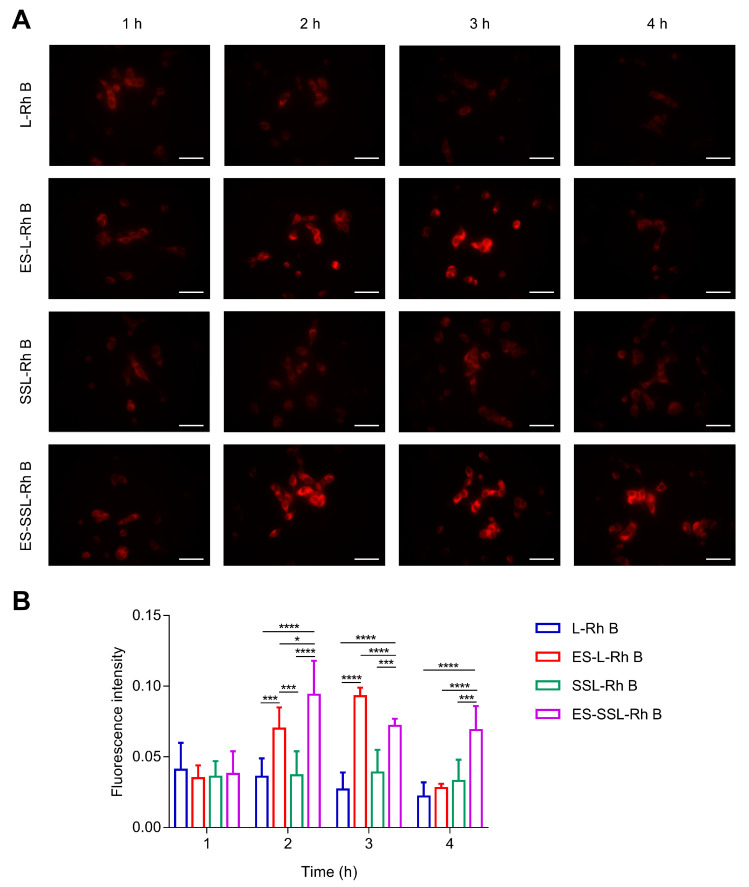
In vitro cellular uptake study. (**A**) Cellular uptake of Rh B-loaded liposomes in A549 cells determined using fluorescence microscopy. Scale bar: 50 μm. Original magnification: ×200. (**B**) Quantitative analysis of fluorescence intensity using Image J, * *p* < 0.05, *** *p* < 0.001, **** *p* < 0.0001.

The intracellular uptake efficiency of the liposomes was assessed to determine targeted ability on A549 cells using fluorescence microscopy. Rh B was used as a fluorescent dye instead of GEM for the cell uptake study. As shown in Figure 6A, ES-SSL-Rh B exhibited the highest fluorescence intensity in the whole cellular uptake process compared with the other groups, and the fluorescence intensity reached the highest intensity at 2 h. Meanwhile, the ES-L-Rh B group also presented obvious red fluorescence which was slightly lower than that of ES-SSL-Rh B, but significantly higher than of the L-Rh B and SSL-Rh B groups. For L-Rh B and SSL-Rh B, the fluorescence intensity was weak because without an ES-targeted fragment, they could not enter the cell through receptor-mediated endocytosis. According to the above results, it could be easier for liposomes to enter into A549 cells by modifying ES. Furthermore, the ES-SSL-Rh B group still maintained a strong fluorescence intensity at 4 h. However, the fluorescence intensity was significantly reduced at 4 h in ES-L-Rh B group. This could be caused by the addition of a PEG fragment, which improved the stability of the liposome and prolonged the time of cellular uptake. It can also be seen from the quantization results in Figure 6B that liposomes modified with ES-targeting fragments, including ES-L-Rh B and ES-SSL-Rh B, significantly increased intracellular fluorescence. PEGylated liposomes could maintain a higher fluorescence intensity for a longer period of time.

### 3.6. Endocytosis Mechanism

**Figure 7:**
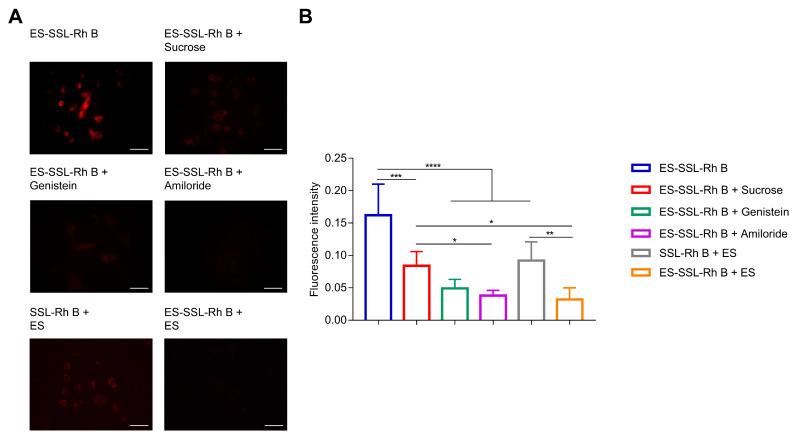
In vitro endocytosis mechanism study. (**A**) Cellular uptake of ES-SSL-Rh B in A549 cells determined using fluorescence microscopy after pretreating with endocytosis inhibitors and ES. Scale bar: 50 μm. Original magnification: ×200. (**B**) The result of fluorescence intensity quantified using Image J, * *p* < 0.05, ** *p* < 0.01, *** *p* < 0.001, **** *p* < 0.0001.

In order to further explore the endocytosis mechanism of ES-SSL, A549 cells were pretreated with ES and three kinds of endocytosis inhibitors, including sucrose, genistein, and amiloride, to observe the changes in ES-SSL-Rh B uptake. ES, as a competitive inhibitor, could block the ER on the surface of A549 cells. As shown in Figure 7A, after the cells were pretreated with ES, the fluorescence intensity of the SSL-Rh B group was significantly higher than that of the ES-SSL-Rh B group, and the fluorescence of the SSL-Rh B group did not decrease significantly, indicating that the addition of ES did not affect the uptake of SSL-Rh B. However, the uptake of ES-SSL-Rh B in A549 cells pretreated with ES decreased significantly, indicating that the prepared ES-SSL-Rh B accumulate intracellularly via ER. ER played an important role in the internalization of ES-SSL-Rh B. Sucrose, which is clathrin-coated pit blocker, was applied to explore the uptake of ES-SSL-Rh B by clathrin-mediated endocytosis [45]. Genistein, a caveolae inhibitor, plays a role in inhibiting endocytosis through caveolin [46]. Na+/H+ exchange is required for macropinocytosis, and amiloride can specifically inhibit this process, which was used to block the macropinocytosis pathway [47]. Figure 7A shows that the control group with ES-SSL-Rh B alone showed a strong red fluorescence intensity. However, after the addition of inhibitors, the fluorescence intensities all decreased to a certain extent, which indicated that these three pathways all participated in the endocytosis of ES-SSL-Rh B. Among them, the red fluorescence of the amiloride group had decreased the most, indicating that the uptake of A549 cells on ES-SSL-Rh B might depend much more on the macropinocytosis. The genistein pretreated group also reduced the uptake of A549 cells, indicating that caveolae-mediated endocytosis was also the main way for ES-SSL-Rh B to enter the cells. Compared with macropinocytosis and caveolae-mediated endocytosis, the clathrin-mediated endocytosis pathway contributed less to the internalization of ES-SSL-Rh B. As shown in Figure 7B, after pretreatment with inhibitors, the fluorescence intensities were 1.93, 3.31, and 4.26 times lower than that of the ES-SSL-Rh B group, respectively. Meanwhile, the blocking of the ER resulted in a fluorescence intensity 5.06 times lower than that of the ES-SSL-Rh B group.

### 3.7. In Vivo Tumor-Targeting Study

**Figure 8:**
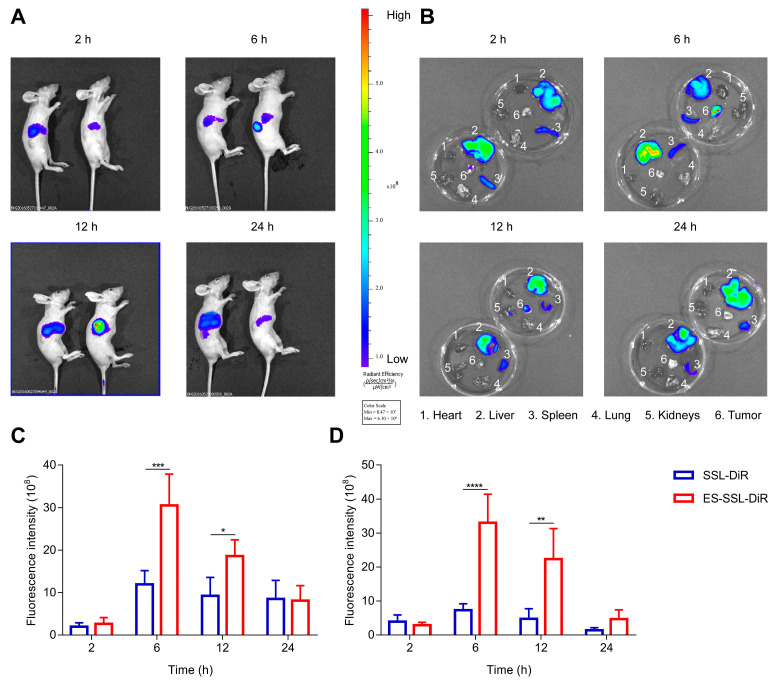
In vivo tumor-targeting study. (**A**) Fluorescence photograph of mice injected with SSL-DiR and ES-SSL-DiR in vivo (left: SSL-DiR group, right: ES-SSL-DiR group). (**B**) Fluorescence photograph of the ex vivo main organs and tumor from mice injected with DiR liposomes (left: SSL-DiR group, right: ES-SSL-DiR group). (**C**) Fluorescence intensity at tumor site in vivo, * *p* < 0.05, *** *p* < 0.001. (**D**) Fluorescence intensity of the ex vivo tumor, ** *p* < 0.005, **** *p* < 0.0001.

For a nano-delivery carrier, the drug should effectively enrich in the tumor to achieve the desired therapeutic effect. The tumor-targeting of different formulations was investigated in BALB/c nude mice using DiR-labeled liposomes. The fluorescence images were taken for 24 h using an IVIS spectrum. As shown in Figure 8A, the red fluorescence in tumors gradually rose and then decreased for both SSL-DiR and ES-SSL-DiR formulations. In the tumor-bearing mice injected with ES-SSL-DiR, a strong fluorescence intensity was observed, reaching the peak intensity at 6 h. The fluorescence in the ES-SSL-DiR group had almost disappeared at 24 h. However, the SSL-DiR group, which lacked tumor targeting, exhibited poor accumulation at the tumor site, in which the fluorescence intensity was relatively weak compared with that of the ES-SSL-DiR group. This result indicated that it was difficult for the SSL-DiR to target the tumor site without the help of the ES-targeting fragment. The modification of ES on liposomes played an important role in enhancing tumor targeting in vivo by receptor–ligand interactions. In addition, the tumors and major organs were harvested for ex vivo fluorescence imaging after imaging in vivo. As shown in Figure 8B, ex vivo fluorescence distribution for the tumor site was also stronger in the ES-SSL-DiR group than in SSL-DiR. The quantitative results are shown in Figure 8C,D. Quantization results also showed that the fluorescence intensity of ES-SSL-DiR in the tumor was significantly higher than that of the SSL-DiR group, and the fluorescence intensity of ES-SSL-DiR in vivo and ex vivo was 2.6 and 4.5 times higher than that of SSL-DiR at 6 h, respectively, which further indicated that ES-SSL-DiR could improve the enrichment of GEM in tumors.

### 3.8. In Vivo Antitumor Efficacy

**Figure 9:**
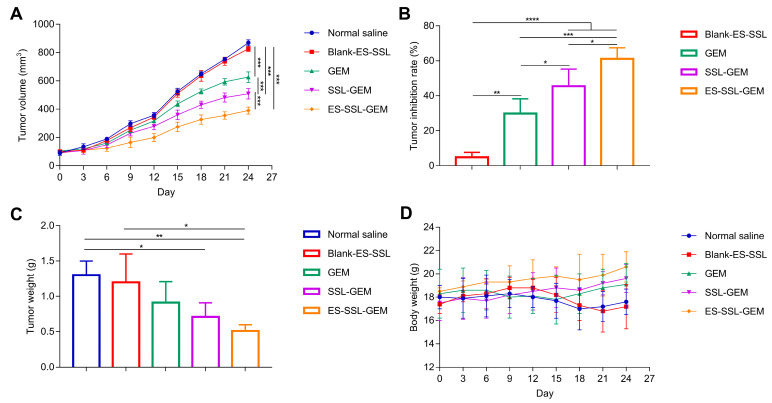
In vivo antitumor effect of ES-SSL-GEM (n = 4). (**A**) Tumor growth curves of BALB/c nude mice after GEM formulations treatment. (**B**) Tumor inhibition rate and (**C**) the weight of the tumor after the experiment. (**D**) The body weight change curves after injection of different formulations. * *p* < 0.05, ** *p* < 0.01, *** *p* < 0.001, **** *p* < 0.0001.

To evaluate the antitumor efficiency of ES-SSL-GEM in vivo, different GEM formulations were administered into tumor-bearing BALB/c nude mice via the tail vein. The body weights and tumor volumes of the athymic mice were continuously monitored for 24 days. As shown in Figure 9A, the tumor volume curve of the normal saline group increased rapidly. After injecting Blank-ES-SSL, the growth trend of tumor volume was consistent with the normal saline group. As shown in Figure 9B, the TIR of the Blank-ES-SSL group was only 4.8%, indicating that the blank formulation itself had no inhibitory influence on tumor growth. Other GEM and GEM formulation groups showed visible inhibition of tumor growth. Although free GEM had a certain inhibitory effect, the extent of tumor suppression was poor. The inhibitory effect of SSL-GEM and ES-SSL-GEM on tumor growth was better than that of the free GEM group. This result was mainly because the liposome formulations had a protective effect on GEM and could passively target the tumor site [48]. Meanwhile, due to the addition of long-term fragments, SSL-GEM and ES-SSL-GEM could avoid phagocytosis by the RES, extending the drug’s blood circulation time and increasing the GEM content in the tumor site so that the anti-tumor ability was enhanced [49]. On the other hand, the ES-SSL-GEM group showed the lowest tumor volume and the strongest inhibitory rate on A549 tumor growth. The TIR of ES-SSL-GEM reached approximately 61.1%, which was 2.05 and 1.34 times higher than that of GEM and SSL-GEM, which had TIRs of 29.8% and 45.5%, respectively. It could also be seen from the result shown in Figure 9C that ES-SSL-GEM presented the smallest tumor weight. All of these results were attributed to the modification of ES-targeted fragments, which could improve the tumor-specific targeting ability and increase the concentration of GEM in the tumor sites to achieve a stronger antitumor efficacy. In addition, the body weight of the mice was used to evaluate the preliminary safety. As shown in Figure 9D, after treatment with different formulations, the body weights did not decrease obviously, suggesting that ES-SSL-GEM is relatively safe.

## 4. Discussion

Compared with free drugs, the application of functional nanocarriers as drug delivery strategies in cancer therapy has attractive advantages, so its development has been widely studied. Some of the benefits include improved distribution of the drug in the body, so that more drugs accumulate in the tumor, which would bring a reduction in the administered dose and thus may lead to reduced side effects [50]. Although the FDA has approved many nanotherapeutic agents for clinical use, liposome formulation is the only nanotherapeutic agent to date that has shown better efficacy than its small-molecule counterpart in a clinical setting [51]. In this study, we designed a GEM-loaded ER-targeted long-acting liposome (ES-SSL-GEM) for the treatment of NSCLC, with the expectation that it will improve tumor targeting and lead to better therapeutic effects.

Many researchers have extensively developed GEM-loaded nanocarriers for cancer treatment. The particle size of ES-SSL-GEM constructed in this study was 131.20 nm. This was consistent with the size of the GEM liposomes modified with antibodies (135 nm) prepared by Yang et al., and all of them were spherically shaped [52]. As stated in the literature, smaller liposomes can be more effectively internalized by endocytic vesicles and can be injected intravenously [53]. In the characterization results, the EE of GEM reached 56.35%, which was slightly higher than that of the GEM and cisplatin co-loaded liposomes prepared by Liu et al., whose EE was 47.7% [54]. It was concluded that ES-SSL-GEM had the advantage of a high EE. In addition, ES-SSL-GEM showed a slower release after PEG modification, which could avoid a large number of GEM being released into the blood circulatory system so that more GEM could reach the tumor [55].

Cell viability assays showed that Blank-ES-SSL was not significantly cytotoxic to non-cancer cells, which may prove that the preparation itself is not toxic. However, when ES-SSL was loaded with GEM, it demonstrated a stronger inhibitory effect on A549 cells or tumors in vitro and in vivo compared with the SSL-GEM group. At the same time, ES-SSL uptake by A549 cells improved, and the tumor enrichment significantly increased. At present, folic acid receptors (FARs), sigma receptors, transferrin receptors (TfRs), EGFR, urokinase plasminogen activator receptors (uPARs), etc., which are overexpressed on the surface of lung cancer cells, have been applied in targeted drug delivery for lung cancer [56]. For example, FAR β-targeted pH-sensitive liposomes containing docetaxel prepared by Park et al. [57] and EGFR-targeted theranostic liposome co-loaded with DNA-biodots and etoposide reported by Jha et al. [58] demonstrated stronger tumor suppression effects for NSCLC. The ES-SSL-GEM prepared in this study, which targeted ER, could also improve the efficacy of free GEM in the treatment of NSCLC. However, the application of ER in targeted preparations for NSCLC has not been reported. Moreover, studies on endocytosis mechanisms have also verified that the targeting ability of ES-SSL needs to be mediated by ER. Multiple endocytic pathways were involved in the uptake of ES-SSL-GEM. Yan Nan also reported that drug-loaded particles were mainly absorbed by cells through endocytosis [59].

## 5. Conclusions

In this work, we successfully prepared estrone-modified GEM-loaded PEGylated liposomes (ES-SSL-GEM) for lung cancer treatment. The prepared liposomes displayed a suitable particle size and high encapsulation efficacy. ES-SSL-GEM showed a sustained and slow release behavior via the modification of the PEG fragment. The targeting studies suggested that ES-SSL-GEM enhanced the active targeted ability. In addition, specific ER-mediated endocytosis was the main pathway to increase cellular uptake of liposomes. Importantly, compared with SSL-GEM, ES-SSL-GEM exhibited more efficient suppression of A549 cells and tumor growth. In conclusion, through the dual modifications of PEG fragments and ES-targeting fragments, ES-SSL-GEM was able to achieve a better treatment effect on lung cancer. This will provide an effective preparation to treat lung cancer.

## Figures and Tables

**Table 1:** Particle size, PDI, zeta potential, and EE of GEM liposome formulations (n = 3).

Formulation	Particle Size (nm)	PDI	Zeta Potential (mV)	EE (%)
L-GEM	118.80 ± 0.05	0.14 ± 0.01	−25.67 ± 0.61	48.27 ± 3.61
ES-L-GEM	120.10 ± 0.16	0.14 ± 0.01	−24.97 ± 0.34	48.90 ± 1.73
SSL-GEM	129.60 ± 0.68	0.19 ± 0.01	−10.04 ± 0.56	55.93 ± 2.98
ES-SSL-GEM	131.20 ± 0.62	0.20 ± 0.01	−9.73 ± 0.41	56.35 ± 1.38

**Table 2:** IC_50_ values (ng/mL) of different GEM formulations on A549 cells (n = 3).

Formulation	24 h IC_50_	48 h IC_50_	72 h IC_50_
GEM	25.98 ± 0.69 *	22.79 ± 0.61 *	19.52 ± 0.52 *
L-GEM	23.32 ± 0.89 *	20.41 ± 0.51 *	6.89 ± 0.43 *
ES-L-GEM	21.49 ± 1.49 *	8.87 ± 1.52 *	1.05 ± 0.85
SSL-GEM	10.39 ± 2.45	5.02 ± 1.45	0.22 ± 0.07
ES-SSL-GEM	8.24 ± 0.31	3.56 ± 0.48	0.09 ± 0.02

Note: * *p* < 0.05, compared with ES-SSL-GEM group.

## Data Availability

Not applicable.
